# Serological Markers of Sand Fly Exposure to Evaluate Insecticidal Nets against Visceral Leishmaniasis in India and Nepal: A Cluster-Randomized Trial

**DOI:** 10.1371/journal.pntd.0001296

**Published:** 2011-09-13

**Authors:** Kamlesh Gidwani, Albert Picado, Suman Rijal, Shri Prakash Singh, Lalita Roy, Vera Volfova, Elisabeth Wreford Andersen, Surendra Uranw, Bart Ostyn, Medhavi Sudarshan, Jaya Chakravarty, Petr Volf, Shyam Sundar, Marleen Boelaert, Matthew Edward Rogers

**Affiliations:** 1 Banaras Hindu University, Varanasi, India; 2 London School of Hygiene and Tropical Medicine, London, United Kingdom; 3 Institute of Tropical Medicine, Antwerp, Belgium; 4 BP Koirala Institute of Health Sciences, Dharan, Nepal; 5 Charles University, Prague, Czech Republic; 6 University of Copenhagen, Copenhagen, Denmark; National Institutes of Health, United States of America

## Abstract

**Background:**

Visceral leishmaniasis is the world' second largest vector-borne parasitic killer and a neglected tropical disease, prevalent in poor communities. Long-lasting insecticidal nets (LNs) are a low cost proven vector intervention method for malaria control; however, their effectiveness against visceral leishmaniasis (VL) is unknown. This study quantified the effect of LNs on exposure to the sand fly vector of VL in India and Nepal during a two year community intervention trial.

**Methods:**

As part of a paired-cluster randomized controlled clinical trial in VL-endemic regions of India and Nepal we tested the effect of LNs on sand fly biting by measuring the antibody response of subjects to the saliva of *Leishmania donovani* vector *Phlebotomus argentipes* and the sympatric (non-vector) *Phlebotomus papatasi*. Fifteen to 20 individuals above 15 years of age from 26 VL endemic clusters were asked to provide a blood sample at baseline, 12 and 24 months post-intervention.

**Results:**

A total of 305 individuals were included in the study, 68 participants provided two blood samples and 237 gave three samples. A random effect linear regression model showed that cluster-wide distribution of LNs reduced exposure to *P. argentipes* by 12% at 12 months (effect 0.88; 95% CI 0.83–0.94) and 9% at 24 months (effect 0.91; 95% CI 0.80–1.02) in the intervention group compared to control adjusting for baseline values and pair. Similar results were obtained for *P. papatasi*.

**Conclusions:**

This trial provides evidence that LNs have a limited effect on sand fly exposure in VL endemic communities in India and Nepal and supports the use of sand fly saliva antibodies as a marker to evaluate vector control interventions.

## Author Summary

Visceral leishmaniasis (VL), also known as kala azar, is one of the major public health concerns of the Indian subcontinent, caused by *Leishmania donovani* transmitted by the bite of the sand fly *Phlebotomus argentipes*. To date, Indoor Residual Spraying (IRS) campaigns have been unable to control the disease. This makes Long-lasting insecticidal nets (LNs) an attractive alternative or complement to IRS. Therefore, it is important to assess the extent that LNs reduce bites from *P. argentipes*. When female sand flies bite they require their saliva to efficiently bloodfeed. For humans and animals alike, the host' immune response against components of sand fly saliva can be used as a marker of exposure to the vector. Here we describe how comprehensive coverage of LNs in trial communities over two years reduced antibody levels to the saliva of *P. argentipes* and *P. papatasi* (a man-biting sand fly that co-exists with *P. argentipes* but does not transmit VL) sand flies by 9–12% compared to communities without LNs. Our results demonstrate that the large-scale distribution of LNs did not confer significant additional protection against sand fly bites in VL-endemic regions of India and Nepal and questions the indoor transmission of *L. donovani* in these regions.

## Introduction

Visceral leishmaniasis (VL or kala azar) is a vector-borne parasitic disease with a fatal outcome if untreated. It is estimated that a large proportion of the annual 500,000 cases and 60,000 deaths occur in poor rural communities of the Indian subcontinent [1]. In these regions VL is exclusively caused by *Leishmania donovani*, transmitted by the bite of female *Phlebotomus argentipes* sand flies, an opportunistic blood feeding sand fly [2]. *Phlebotomus papatasi*, a man-biting sand fly sympatric with *P. argentipes* throughout the Indian subcontinent, does not transmit *L. donovani*, but is the Old World vector of zoonotic cutaneous leishmaniasis in much of Northern Africa and the Middle East [3]. Since there is no vaccine for VL, control measures depend on early case-detection, treatment and reduction in transmission through vector control measures. Current control of VL vectors in the Indian subcontinent is based on indoor residual spraying (IRS) of insecticides. Despite these efforts, the current strategy is failing to control VL in these regions [4]. Because *L. donovani* transmission is anthroponotic, and humans represent the only proven reservoir of infection, attention is being focused on the use of insecticide treated nets (ITNs), specifically, long-lasting insecticidal nets (LNs) to replace or compliment IRS.

Village-wide distribution of LNs have shown to significantly reduce indoor *P. argentipes* density by 25% [5], 44% [6] and 60% [7] in the Indian subcontinent. The variation observed in the effect of LN on *P. argentipes* density could be related to differences in experimental designs, vector behavior or insecticide susceptibility in Bangladesh, India and Nepal. Nevertheless the results of the first large-scale randomized controlled trial of the effectiveness LN to prevent VL in India and Nepal, indicate that LNs seem to have a small and not significant effect on the risk of *L. donovani* infection and clinical disease in VL endemic communities. During this trial the risk for *L. donovani* infection, measured by means of Direct Agglutination Test (DAT), was reduced by 10% in clusters using LNs compared to controls [8]. Therefore, a tool to measure exposure to the VL vector will allow us to bridge a gap between the entomological and clinical results observed. The most direct way of doing this is by recording the numbers of bites individuals receive; however, since human landing catches are unethical for VL (VL is fatal with no effective prophylaxis) there are only a handful of studies reporting biting or landing rates of *P. argentipes* in VL foci [9]–[12]. An alternative method is required.

Sand flies rely on the vasodilatory and anti-haemostatic properties of their saliva to obtain blood for egg production and consequently salivate into the host' skin with each bite. The relationship between the levels of antibodies to arthropod saliva, vector exposure and risk of infection has been demonstrated for a variety of vector-host models. Mosquito and tick saliva were associated with the risk of contracting malaria [13] and Lyme disease [14], respectively, and *Triatoma infestans* saliva was used as a marker for vector infestation in domestic animals [15]. Sand fly saliva has been shown to be highly immunogenic for both humans and animals alike [16]–[17], and experimental studies have shown that the level of antibodies to salivary proteins are proportional to the number of bites or amount of saliva injected [18]. This provides an opportunity to develop a versatile tool to understand the transmission, epidemiology and risk of leishmaniasis and evaluate vector intervention programs.

In Angola antibodies to the saliva of *Anopheles gambiae* was successfully used to evaluate the efficacy of LNs against malaria [19]. Recently, we developed a single saliva-based ELISA to measure human antibodies to *P. argentipes* and *P. papatasi* in VL-endemic areas [20]. An entomological survey of Indian and Nepalese households was used to assess the use of this ELISA as a tool to measure vector exposure. Indoor CDC light trap captures, used as proxy for sand fly exposure, were correlated to sand fly saliva antibodies in people. Similarly, in a small scale study of VL patients in Muzzafarpur, an endemic district of VL in India, we found that admission to hospital – thus protecting patients from sand fly bites for 30 days – resulted in a significant drop in antibodies to *P. argentipes* and *P. papatasi* saliva, which quickly rose again when treated patients returned to their villages and were re-exposed. To date, sand fly salivary antibodies have not been used to evaluate vector intervention programs at the community level. In the current study we screened sera from people given Deltamethrin-impregnated bednets, or not, to sleep under to assess their levels of anti-sand fly salivary antibodies over two years.

The objective of this study was to detect antibodies to *P. argentipes* and *P. papatasi* saliva to determine the effect of LNs on vector and non-vector sand fly exposure in VL-endemic villages of India and Nepal.

## Materials and Methods

### Study population

The blood samples included in this study are a subset of the samples collected in a large-scale, randomised controlled trial on the effectiveness of comprehensive LN distribution to prevent VL in the Indian subcontinent (KALANET, ClinicalTrials.gov CT-2005-015374). The study design is briefly described here. In May 2006, 26 VL endemic clusters with over 20,000 inhabitants were selected in India (n = 16) and Nepal (n = 10) based on their VL incidence from 2003 to 2005. The study clusters were matched by country, population size and pre-intervention VL incidence and randomly allocated to intervention or control groups, 13 clusters per arm. All households in the intervention group received Deltamethrin coated LNs (PermaNet 2.0) at baseline (November-December 2006). Enough LNs were distributed to ensure all households members slept under a net.

For the main trial outcome, we collected finger prick blood samples at baseline and at 12 and 24 months post-intervention from all participants over 2 years of age. Incident *L. donovani* infections were determined by Direct Agglutination Test (DAT). Further details on the study design and on the effect of LNs on indoor sand fly density, *L. donovani* infection and VL are described elsewhere [5], [8].

For this study, 15 to 20 individuals were selected in each study cluster in October 2006. The individuals were randomly selected among all the inhabitants in each cluster using the data collected in a demographic survey conducted in July 2006. Only individuals above 15 years of age were eligible these participants were asked to provide a larger amount of blood (3 ml) by vein puncture at baseline, 12 and 24 months post-intervention. The sera obtained by centrifugation were identified with the individual ID and kept at −20°C until the laboratory analyses were conducted. Information on the age, gender, VL history, DAT titre at baseline, malnutrition and Socio-Economic Status (SES) were available for all participants. The methods used to evaluate the malnutrition and SES are described in detail elsewhere [21].

### Saliva preparation

Salivary gland lysate (SGL) of colonised *P. argentipes* and *P. papatasi* sand flies (Charles University, Prague, Czech Republic) was obtained as described previously [16]–[17], [20]. Salivary glands were dissected from female flies maintained on sucrose solution *ad libitum* at five days old post-emergence. SGL was lyophilized and reconstituted in its original volume of distilled water for 1 hr at room temperature (25°C) before use.

### Laboratory analyses

Pre-adsorption of sera against *P. papatasi* saliva significantly improves the specificity of the *P. argentipes* ELISA by reducing the levels of cross-reaction [20]. This is achieved by reducing the amount of antibodies which commonly recognise salivary antigens of both these sand flies. To do this, 50 ng *P. papatasi* SGL in bicarbonate buffer (pH 9.6) was coated in each well of microtiter plates (maxisorp, Nunc) at 4°C overnight. After washing 4 times (PBS- 0.05% Tween 20 (PBS-T) Fluka, Sigma), plates were blocked with 5% bovine serum albumin in PBS-T for 2 hr at 37°C. After washing, 1∶50 diluted human sera in PBS-T were added and incubated overnight at 4°C (the *P. papatasi* pre-adsorption step. Simultaneously another plate was coated with SGL of *P. argentipes* (50 ng/well) at 4°C overnight. The next day after washing and blocking of the *P. argentipes* plate, sera were transferred from the *P. papatasi* plate and incubated at 37°C for 2 hr. From this point both plates were processed in parallel. Plates were incubated with biotinylated goat anti-human IgG (1∶1000 in PBS-T, Sigma) for 1 hr at 25°C, washed and incubated with streptavidin-conjugated alkaline phosphatase (1∶1000 dilution in PBS-T, Sigma) for a further 1 hr at 25°C. To develop the reaction substrate (paranitrophenylphosphate, 1 mg/ml, Sigma) was added and the optical density (OD) measured at 405 nm using a Spectramax 190 ELISA plate reader after 20 minutes incubation in the dark. To minimise day to day variation in ELISA performance three sera from the same individual collected over the entire trial (baseline; 12 and 24 months follow-up samples) were processed in the same plate.

Cut offs for positive *P. argentipes* and *P. papatasi* ELISA were determined as the average OD values plus two standard deviations of 9 Indian non-endemic controls (NEC) from urban, non-VL areas of Western Uttar Pradesh [20].

### Statistical analyses

Individual and geometric mean ELISA OD per immunological survey: baseline (Nov-Dec 2006), 12 and 24 months follow-up; and intervention group (LN and control clusters) were plotted and tabulated.

A random effect linear regression model was used to estimate the effect of LN on the log transformed ELISA OD at 12 and 24 months. The following model was applied: 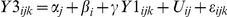



Where the outcome Y3_ijk_ is the log-transformed OD at 24 months for person *k* in cluster *j* and treatment arm *i*. And α_j_ is a fixed pair effect to take the matching into account, β_i_ is the intervention effect, *γ* is the effect of the log-transformed baseline value Y1, U_ij_ is a random cluster effect assumed normally distributed with mean 0 and variance σ_B_^2^ (the between cluster variation within matched pairs) and ε_ijk_ the individual measurement error also assumed normal with mean 0 and variance σ_w_^2^ (variation between individuals within same cluster). The main parameter of interest is β_i_, which measures the mean difference in log OD at 24 months for two persons from the same pair, with the same baseline OD, one from the intervention cluster and the other from the control cluster. The fit of the model was checked by residual plots. An analogous model was used to study the log-transformed ELISA OD result at 12 months.

In separate analyses those individuals with anti-*P. argentipes* or anti-*P. papatasi* OD values below the cut offs or no records at baseline were removed to increase the sensitivity of the data [22]. The data were analysed in Stata 11 (StataCorp LP, College Station, TX, USA).

### Ethical issues

Written informed consent was obtained from each participant or their guardian for those under 18 years old. Ethical approval was obtained from the Institutional Review Boards (IRB) of the B. P. Koirala Institute of Health Sciences, Nepal; the Institute of Medical Sciences Banaras Hindu University, India and the Institute of Tropical Medicine, Antwerp, Belgium.

## Results

### Study population

**Figure 1:**
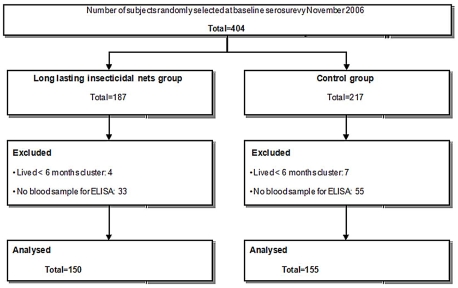
Study population flow chart. Number of individuals initially enrolled in the study and number of subjects excluded or lost to follow-up (no blood samples available) per study (intervention and control) group.

A total of 305 individuals were included in the study, 68 participants provided two blood samples and 237 gave three samples. As shown in the study population flow chart (Figure 1), more individuals were excluded or lost to follow-up in the control (n = 62) than in the intervention (n = 37) group. However, there were no significant differences between both groups (Table 1). The study population characteristics are summarized in Table 2. The number of subjects per group was well balanced: 150 and 155 in intervention and control groups respectively. There were however some differences, the intervention group had more past VL cases or people living in households with a VL case in the previous 24 months. The control group had fewer individuals moderately or severely malnourished. When the samples were restricted to those with positive ELISA results at baseline, the number of samples was reduced in both groups but especially in the controls and for *P. papatasi* (Table 2).

**Table 1:** Characteristics of individuals excluded and lost to follow-up.

	Lost to follow-up	
	Control	Intervention
Total Individuals	62	37
Mean age (SD)	27.5 (7.7)	26.4 (6.9)
No. males (%)	39 (63%)	23 (62%)
Percentage of DAT positive at baseline^1^ (n/N)	17% (10/57)	17% (6/36)
No. individuals with past history of VL (%)	6 (9.7%)	3 (8.1%)
Mean SES indicator^2^ (SD)	2.1 (1.6)	1.8 (1.4)
Percentage of individuals with Moderate or Severe Malnutrition^3^ (n/N)	5.2% (3/57)	2.8% (1/36)
No. Individuals living in houses with at least one VL case in past 24 months (%)	6 (9.7%)	5 (13.5%)

1 Direct Agglutination Test (DAT) titre ≥1∶1600. ^2^Socio-Economic Status indicator calculated as detailed in Singh et al[21]. ^3^Nutrition status calculated as detailed in Singh et al [21].

**Table 2:** Study population characteristics.

	All available samples		Restricted *P. argentipes* 1		Restricted *P. papatasi^2^*	
	Control	Intervention	Control	Intervention	Control	Intervention
Total Individuals (range per cluster)	155 (7–17)	150 (6–17)	72 (2–14)	91 (4–12)	26 (1–5)	47 (1–9)
Mean age (SD)	28.2 (7.6)	28.3 (7.5)	27.4 (8.5)	27.6 (7.7)	25.9 (7.0)	28.5 (7.3)
No. males (%)	48 (31.0)	63 (42.0)	25 (34.7)	38 (41.8)	8 (30.8)	20 (42.6)
No. DAT positive at baseline^3^ (%)	24 (15.5)	27 (18.0)	14 (19.4)	17 (18.7)	4 (15.4)	8 (17.0)
No. individuals with past history of VL (%)	8 (5.2)	13 (8.7)	3 (4.2)	9 (9.9)	1 (3.8)	1 (2.1)
Mean SES indicator^4^ (SD)	2.2 (1.4)	1.8 (1.4)	2.3 (1.4)	1.8 (1.4)	2.0 (1.3)	1.8 (1.4)
No. Individuals with Moderate or Severe Malnutrition^5^ (%)	12 (7.7)	18 (12.0)	1 (1.4)	8 (8.8)	0 (0)	4 (8.5)
No. Individuals living in houses with at least one VL case in past 24 months (%)	11 (7.1)	14 (9.3)	6 (8.3)	7 (7.7)	2 (7.7)	3 (6.4)

1 Excluding records with no ELISA results or OD for *P. argentipes* below 0.9 at baseline (n = 163). ^2^Excluding records with no ELISA results or OD for *P. papatasi* below 1.8 at baseline (n = 73). ^3^Direct Agglutination Test (DAT) titre ≥1∶1600. ^4^Socio-Economic Status indicator calculated as detailed in Singh et al [21]. ^5^Nutrition status calculated as detailed in Singh et al [21].

### Sand fly saliva ELISA results

**Figure 2:**
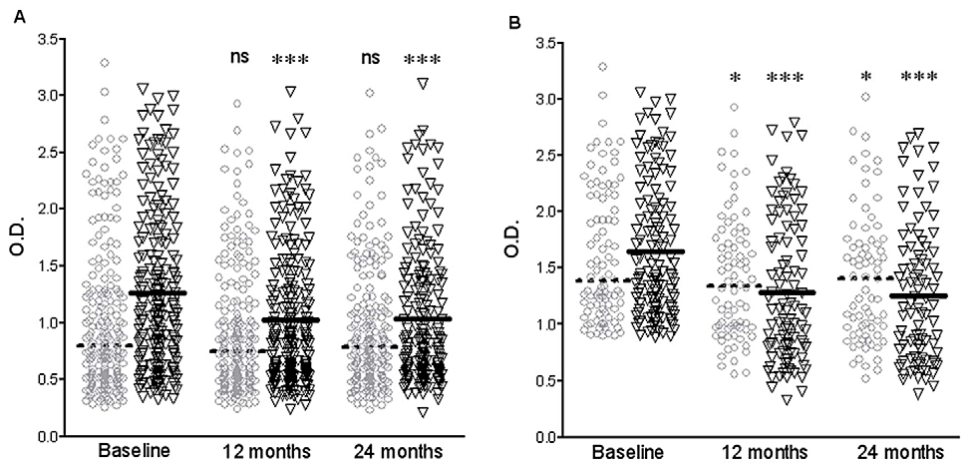
Effect of LNs on human exposure to *P. argentipes* and *P. papatasi* sand flies. Individual ELISA Optical Density (OD) per immunological survey (baseline, 12 and 24 months follow-up) for intervention (long-lasting insecticidal nets, LN – black triangles) and control clusters (grey circles), for *Phlebotomus argentipes* (Panel A) and *P. papatasi* (Panel B). The geometric means ELISA OD are represented as a solid line for LN and dotted line for control groups. Results represent all the samples available (n = 305). The Mann Whitney t-test was used to compare 12 and 24 month follow-up samples compared to their corresponding baseline values, asterisks denote statistical significance (*, P<0.05; **, P<0.005; *, P<0.005; ns, not significant P>0.05).

At baseline the intervention group had higher geometric mean ELISA OD than control group, both for *P. argentipes* (1.10 vs. 0.86) and *P. papatasi* (1.21 vs. 1.05) (Table 3 & Figure 2). The geometric mean ELISA OD for *P. argentipes* and *P. papatasi* declined in the group using LN at 12 and 24 months but remained more or less constant in the control group at the same time points (Figure 2). Analysing the data with a random effect linear regression model found that intervention was significantly associated (p-value<0.05) with ELISA results at 12 months for *P. argentipes* and *P. papatasi* and at 24 months for *P. papatasi* only (Table 3). For *P. argentipes* the geometric mean of ELISA OD was on average 12% reduced at 12 months (effect 0.88; 95% CI 0.83–0.94) and 9% at 24 months (effect 0.91; 95% CI 0.80–1.02) in the intervention group compared to control adjusting for baseline OD and pair. Similar results were obtained for *P. papatasi*: 11% (effect 0.89) and 9% (effect 0.91) reduction in LN group at 12 and 24 months respectively (Table 3).

**Table 3:** Average anti-saliva antibody response.

	Control			Intervention			Intervention effect	
	No. Samples	GM ELISA OD (IQR)	No. Positive^1^ (%)	No. Samples	GM ELISA OD (IQR)	No. Positive^1^ (%)	Fold change from baseline (95% CI)	p-value
***P. argentipes***								
Baseline	153	0.86 (0.52; 1.33)	72 (47.1)	144	1.10 (0.74; 1.71)	91 (63.2)		
12 months	142	0.80 (0.49; 1.32)	59 (41.5)	144	0.92 (0.59; 1.44)	70 (48.6)	0.88 (0.83; 0.94)	<0.001
24 months	140	0.83 (0.52; 1.26)	61 (43.6)	124	0.88 (0.59; 1.33)	54 (43.5)	0.91 (0.80; 1.02)	0.115
***P. papatasi***								
Baseline	153	1.05 (0.70; 1.57)	26 (17.0)	144	1.21 (0.83; 2.02)	47 (32.6)		
12 months	142	1.05 (0.74; 1.70)	29 (20.4)	144	1.11 (0.78; 1.77)	35 (24.3)	0.89 (0.82; 0.96)	0.002
24 months	140	1.02 (0.70; 1.66)	24 (17.1)	124	1.03 (0.67; 1.64)	27 (21.8)	0.91 (0.84; 0.99)	0.034

Geometric mean (GM) and inter quartile range (IQR) of ELISA Optical Density (OD) per immunological survey (baseline, 12 and 24 months follow-up) and intervention group (LN and control clusters) for *Phlebotomus argentipes* and *P. papatasi*. ^1^Number of samples positive per survey using 0.9 and 1.8 ELISA OD as cut off values for *P. argentipes* and *P. papatasi* respectively. Estimates of the intervention effect at 12 and 24 months adjusting for pair and baseline ELISA OD value. Results were obtained using all samples available (n = 305).

The cut off values obtained for *P. argentipes* and *P. papatasi* were 0.9 and 1.8 ELISA OD respectively. Using these values as a reference, the percentages of positive samples for *P. argentipes* ELISA were reduced from 63.2% to 43.5% and from 47.1% to 43.6% in the intervention and control groups respectively over 24 months (Table 3). For *P. papatasi*, the percentage of positive ELISA samples was not altered after 24 months in the control clusters (17%) but was reduced from 32.6% to 21.8% in the clusters using LN (Table 3).

**Figure 3:**
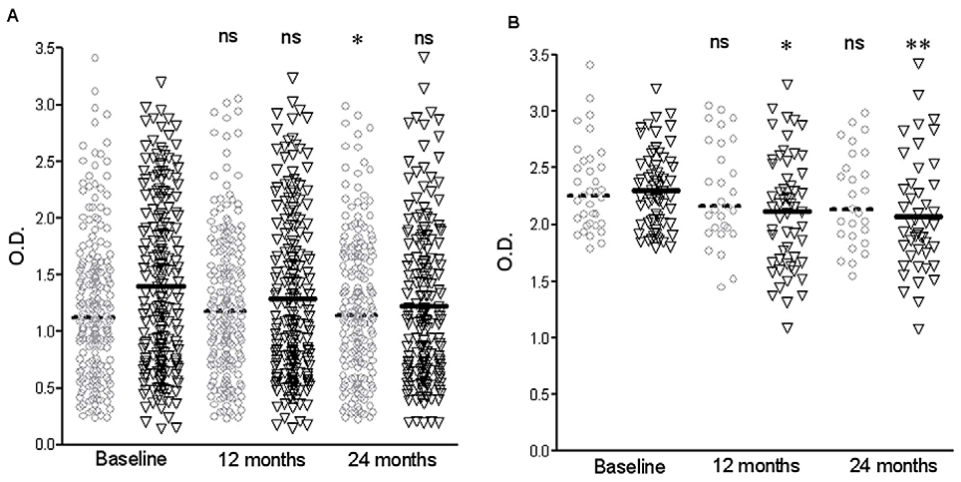
Effect of LNs on sand fly exposure, adjusted for non-endemic controls. Individual ELISA Optical Density (OD) per immunological survey (baseline, 12 and 24 months follow-up) for intervention (long-lasting insecticidal net, LN – black triangles) and control clusters (grey circles), for *Phlebotomus argentipes* (Panel A) and *P. papatasi* (Panel B). Individuals with no ELISA results or below the average non-endemic control OD+2×S.D. cut-off values (0.9 for *P. argentipes* and 1.8 for *P. papatasi*) at baseline were excluded. The geometric means ELISA OD are represented as a solid line for LN and dotted line for control groups. The Mann Whitney t-test was used to compare 12 and 24 month follow-up samples compared to their corresponding baseline values, asterisks denote statistical significance (*, P<0.05; **, P<0.005; *, P<0.005; ns, not significant P>0.05).

When the non-responders at baseline (no ELISA results or OD<cut off) were excluded from the analyses, to improve the sensitivity of the ELISA [18], the geometric means at baseline were equilibrated in both groups and for both sand fly species (Table 4 & Figure 3). The effect of LN on *P. argentipes* exposure was similar to the one observed when all samples were used. The intervention was significantly associated with ELISA results only at 12 months for *P. argentipes*. The geometric mean of ELISA OD was on average reduced by 14% and 14% for *P. argentipes* and 6% and 7% for *P. papatasi* at 12 and 24 months respectively in the intervention group compared to control (Table 4).

**Table 4:** Average anti-saliva antibody response – baseline adjusted.

	Control			Intervention			Intervention effect	
	No. Samples	GM ELISA OD (IQR)	No. Positive^1^ (%)	No. Samples	GM ELISA OD (IQR)	No. Positive^1^ (%)	Fold change from baseline (95% CI)	p-value
***P. argentipes***								
Baseline	72	1.52 (1.15; 2.14)	72 (100)	91	1.54 (1.12; 2.03)	91 (100)		
12 months	59	1.35 (0.97; 1.82)	48 (81.4)	85	1.22 (0.89; 1.72)	62 (72.9)	0.86 (0.78; 0.96)	0.005
24 months	60	1.33 (0.99; 1.75)	49 (81.7)	68	1.13 (0.84; 1.51)	45 (66.2)	0.86 (0.73; 1.01)	0.071
***P. papatasi***								
Baseline	26	2.30 (2.01; 2.50)	26 (100)	47	2.28 (2.04; 2.53)	47 (100)		
12 months	22	2.22 (1.92; 2.75)	18 (81.8)	42	2.07 (1.66; 2.57)	28 (66.7)	0.94 (0.84; 1.06)	0.321
24 months	21	2.23 (1.95; 2.62)	17 (81.0)	30	2.01 (1.75; 2.31)	21 (70.0)	0.93 (0.82; 1.06)	0.278

Geometric mean (GM) and inter quartile range (IQR) of ELISA Optical Density (OD) per immunological survey (baseline, 12 and 24 months follow-up) and intervention group (LN and control clusters) for *Phlebotomus argentipes* and *P. papatasi*. ^1^Number of samples positive per survey using 0.9 and 1.8 ELISA OD as cut off values for *P. argentipes* and *P. papatasi* respectively. Estimates of the intervention effect at 12 and 24 months adjusting for pair and baseline ELISA OD value. Results were obtained excluding records with no ELISA results or OD below the cut off values at baseline.

## Discussion

The results of this study show that *P. argentipes* exposure was reduced by 9 to 12% in people living in villages where LNs were used compared to controls. This reduction is in the same order of magnitude of the effect of LN on *L. donovani* infection observed in the same study clusters in India and Nepal [8]. Even if the use of LN reduced the *P. argentipes* indoor density in the study clusters [5] and seemed to provide some degree of personal protection [8], [23], a significant number of subjects living in intervention clusters had high levels of antibodies against *P. argentipes* after 24 months of LN use (43.5% were ELISA positive). These results could be explained if LN failed to reduce the sand fly abundance as shown in a previous study in the area [24] or by the incorrect use of LN. However, as over 90% of the participants in the intervention clusters use the LN regularly (i.e. over 80% of the nights), they seem to support the theory that a substantial fraction of *L. donovani* transmission occurs outside the house where LNs would not prevent sand fly-human contact [8]. This goes against the traditional narrative that *P. argentipes* predominantly bite at night, and inside houses [9]–[12]. This hypothesis cannot be proved with this study design but it is supported by the trial results as a whole: i.e. similar *L. donovani* infection (5.4% vs. 5.5%) and VL (0.38% vs. 0.40%) rates were reported in both intervention and control clusters [8]. Moreover, *P. argentipes* are known to breed outside households [25], significant numbers of *P. argentipes* captured around households [26]–[27] and about 15–20% of those collected in cattle sheds had fed on humans [28]–[29]. The latter results could be related to the movement of blood fed females but they also suggest that *P. argentipes* are somehow exophagic.

The effect of LNs on *P. papatasi* exposure was similar with a 9–11% reduction in exposure. *Phlebotomus papatasi* is a sand fly sympatric with *P. argentipes* throughout the Indian subcontinent, is also endophilic and highly anthropophilic [30]–[31]. Although it is man-biting, *Phlebotomus papatasi* does not vector *L. donovani*. However, it would appear that LNs protect against the bite of *P. argentipes* and *P. papatasi* equally.

By removing the non-responders we improve the specificity of the saliva ELISA. The adjusted data revealed that the difference in geometric mean of ELISA ODs for *P. argentipe*s shows a greater drop that that observed for *P. papatasi*. This may be due to differences in bloodfeeding or resting behaviour, as hypothesised above. Currently, *P. argentipes* is considered more endophilic than endophagic, often found digesting their bovine bloodmeals within households which have live stock nearby, or are commonly housed in the same building [25], [27]–[29]. In contrast, *P. papatasi* is considered less opportunistic and more endophagic [30]–[31]. If this is the case one would expect a larger drop in ELISA ODs against *P. papatasi* as they would come into contact with the LN, attracted to the sleeping occupant. Recently, Dinesh and colleagues showed that *P, argentipes* from the same areas of India and Nepal as our study were very sensitive to deltamethrin but *P. papatasi* was not compared [32]. Therefore, there remains the possibility of different susceptibilities to this insecticide between different sand fly populations. An alternative hypothesis is that the LNs repelled *P. papatasi*, although no such properties have been reported in the literature.

The results of this study support the use of the sand fly saliva ELISA as a sensitive tool to evaluate vector control intervention. Similar methods have been used to assess the exposure to *Anopheles gambiae* in natural conditions in Senegal [33] and to evaluate the efficacy of ITNs in malaria vector control in Angola [19]. The latter study reported a significant decrease in the antibody response to *An. gambiae* after the introduction of ITNs. However, in contrast to our study, the magnitude of the effect was not assessed, a “before and after intervention” design was used (so there were no concurrent controls) and only 109 samples were analysed [19].

The baseline sand fly saliva antibody values were different between intervention and control groups; people in intervention clusters seemed to have a higher sand fly exposure before the LNs were distributed (Figure 1). This contrasts with the baseline data from the trial which showed that intervention and control clusters had similar indoor *P. argentipes* density [5] and similar population characteristics [8]. This difference may be due to random error as the number of samples per cluster was small (6 to 17 subjects/cluster) and there were some differences between groups at baseline: i.e. more past VL cases in the intervention group (Table 1). Differential dropout between the study groups may have also caused the differences observed at baseline. However, even if there were more individuals lost to follow-up in control group than in the intervention group, the individuals excluded from both groups had similar characteristics (Table 1). To take into account the differences at baseline, the statistical model used to evaluate the impact of LN on sand fly exposure was adjusted for baseline values. Similarly, when the analyses were restricted to positive ELISA results at baseline to increase the sensitivity of the test [22], the baseline values were equilibrated between groups and the effect of LN on *P. argentipes* exposure remained unaltered (14% reduction).

Antibody-based assays to measure vector exposure represents an advance from traditional methods of vector sampling since light traps are not effective in catching bloodfed *P. argentipes* [34] and are unable to measure the human-sand fly contact outside households. It is important that such assays are specific to the vector, sensitive to the number of bites received and responsive to changes in exposure over time [35], [20]. We have previously shown that our saliva-ELISA correlates with indoor *P. argentipes* densities, and pre-adsorption of sera against *P. papatasi* saliva reduced cross-reaction with this non-VL vector, which may lead to false positive results [20]. In the future, recombinant peptides screened from cDNA libraries constructed from *P. argentipes* (and *P. papatasi*) salivary glands will insure against this problem. Despite the drawbacks of using whole saliva as ELISA antigen (labour intensive, costly and time consuming) experimental studies have shown that not all saliva-positive human sera recognize the same protein bands [36], [17], [22]. In this respect, whole saliva has an advantage as it represents all peptides.

In conclusion we demonstrate that the current *P. argentipes* saliva antibody test is a useful tool for the evaluation of vector intervention programmes in human populations from VL-endemic areas. It would appear that LNs have a limited effect on sand fly exposure and combined interventions that address the peri-and intradomestic environment seem the way forward. VL control will require strengthening vector control methods. Rapid case detection and treatment alone may be insufficient to control *L. donovani* transmission if asymptomatic infected individuals play a role in VL epidemiology as a recent mathematical model suggests [37]. More research on the behaviour of *P. argentipes* in relation to *L. donovani* transmission would be prudent to refine future intervention strategies for VL.

## Supporting Information

Checklist S1 CONSORT checklist.
(DOCX)
